# Characterization of the Expressions and m6A Methylation Modification Patterns of mRNAs and lncRNAs in a Spinal Cord Injury Rat Model

**DOI:** 10.1007/s12035-024-04297-z

**Published:** 2024-06-22

**Authors:** Xin Liu, Zhiling Li, Juncheng Tong, Fan Wu, Hui Jin, Kaiqing Liu

**Affiliations:** 1 Shenzhen Eye Hospital, Jinan University, Shenzhen Eye Institute, Shenzhen, 518040 Guangdong China; 2 Department of Traditional Chinese Medicine, The Seventh Affiliated Hospital, Sun Yat-Sen University, Shenzhen, 518107 China; 3 Shenzhen Luohu Hospital Group, The Third Affiliated Hospital of Shenzhen University, Shenzhen, 518000 China; 4 Present Address: National Clinical Research Center for Infectious Diseases, Shenzhen Third People’s Hospital, Shenzhen, 518112 Guangdong China; 5 Research Centre, The Seventh Affiliated Hospital, Sun Yat-Sen University, Shenzhen, 518107 China

**Keywords:** Spinal cord injury, m6A methylation modification, mRNA, lncRNA

## Abstract

Spinal cord injury (SCI) is a serious central nervous system disease with no effective treatment strategy presently due to its complex pathogenic mechanism. N6-methyladenosine (m6A) methylation modification plays an important role in diverse physiological and pathological processes. However, our understanding of the potential mechanisms of messenger RNA (mRNA) and long non-coding RNAs (lncRNA) m6A methylation in SCI is currently limited. Here, comprehensive m6A profiles and gene expression patterns of mRNAs and lncRNAs in spinal cord tissues after SCI were identified using microarray analysis of immunoprecipitated methylated RNAs. A total of 3745 mRNAs (2343 hypermethylated and 1402 hypomethylated) and 738 lncRNAs (488 hypermethylated and 250 hypomethylated) were differentially methylated with m6A modifications in the SCI and sham rats. Functional analysis revealed that differentially m6A-modified mRNAs were mainly involved in immune inflammatory response, nervous system development, and focal adhesion pathway. In contrast, differentially m6A-modified lncRNAs were mainly related to antigen processing and presentation, the apoptotic process, and the mitogen-activated protein kinases (MAPKs) signaling pathway. In addition, combined analysis of m6A methylation and RNA expression results revealed that 1636 hypermethylated mRNAs and 262 hypermethylated lncRNAs were up-regulated, and 1571 hypomethylated mRNAs and 204 lncRNAs were down-regulated. Furthermore, we validated the altered levels of m6A methylation and RNA expression of five mRNAs (CD68, Gpnmb, Lilrb4, Lamp5, and Snap25) and five lncRNAs (XR_360518, uc.393 + , NR_131064, uc.280 − , and XR_597251) using MeRIP-qPCR and qRT-PCR. This study expands our understanding of the molecular mechanisms underlying m6A modification in SCI and provides novel insights to promote functional recovery after SCI.

**Supplementary Information:**

The online version contains supplementary material available at 10.1007/s12035-024-04297-z.

## Introduction

Spinal cord injury (SCI) is a traumatic event that can lead to impairment of motor, sensory, and autonomic function [1]. Primary SCI is commonly caused by direct trauma resulting in immediate hemorrhage and rapid neuronal cell death. This is followed by a secondary injury that induces various cellular inflammatory responses and apoptosis, leading to further expansion of the initial injury [1, 2]. After SCI, the blood-spinal cord barrier is destroyed, leading to immune microenvironment disorder and poor regeneration of the damaged spinal cord. Due to the decreased function of immune cells, patients with SCI exhibit a higher rate of disease infection [3, 4]. Based on the bulk RNA sequencing datasets and scRNA-seq, a study suggests that B2m, Itgb5, and Vav1 in macrophages/microglia may be key therapeutic targets for improving spinal cord function during the subacute phase of SCI. Low-dose decitabine may promote the recovery of neurological function after SCI by regulating the polarization state of macrophages/microglia [5]. However, due to the high complexity of the spinal cord after spinal cord injury and the unclear molecular mechanisms, there is no effective clinical treatment for SCI.

Epigenetic RNA modification is an important conservative post-transcriptional mechanism in which N6-methyladenosine (m6A) is a common type of modification in mammalian cells and is regulated by the interaction of m6A methyltransferases, demethylases, and binding proteins [6, 7]. As the most widely present epigenetic modification of mRNA and non-coding RNA, m6A modification regulates protein expression by influencing mRNA expression, splicing, and translation and participates in the regulation of various diseases, including cancer, cardiovascular diseases, and neurological diseases [8–12]. For example, m6A-modified transcripts have been implicated in the pathophysiological consequences of traumatic brain injury (TBI). Furthermore, TBI-induced brain damage may lead to disturbances in energy homeostasis and mitochondrial dysfunction, which may cause neurodegeneration and altered synaptic plasticity, resulting in cognitive impairment [13]. In addition, owing to the lack of a coding function, m6A modification of long non-coding RNAs (lncRNA) is mainly involved in the occurrence and progression of diseases by influencing the interaction between lncRNAs and RNA or proteins [14, 15].

Based on methylated RNA immunoprecipitation combined with high throughput sequencing or methylated RNA immunoprecipitation combined with microarray analysis technology, several studies have demonstrated that m6A modification of mRNA is involved in SCI development in rat, mice, and zebrafish [16–19]. Moreover, studies have shown that METTL14-mediated m6A modification is involved in spinal cord neuronal apoptosis in SCI [20, 21]. Additional studies have revealed that METTL3 promotes reactive astrocyte proliferation through the m6A modification of YAP1 and improves functional recovery after SCI [22]. However, the role of m6A modification of lncRNAs in SCI rat pathogenesis is unclear.

In this study, we obtained the transcription profiles of m6A-modified mRNAs and lncRNAs in the spinal cord tissues of sham-operated and SCI rats via microarray analysis of immunoprecipitated methylated RNAs and determined the biological pathways involved in m6A methylation in SCI. By combining the changes in mRNA and lncRNA transcription levels after SCI, we further explored the potential relationship between RNA methylation modification and gene expression. The results of the present study will contribute to the elucidation of the pathophysiology of SCI and provide new insights for treatment.

## Materials and Methods

### Rat SCI Model Establishment

Adult female SD rats (*n* = 12; 7–8 weeks old; 220–250 g) supplied by ZhuHai Bestest Biotechnology Co., Ltd., were used to prepare the SCI model. All experimental protocols and animal handling procedures were approved (no. 20210077) by the Animal Care and Ethics Committee of Shenzhen Top Biotech Co., Ltd. The surgical procedures for SCI were performed as previously described [23]. All rats were anesthetized with isoflurane (5% for induction and 1.5% or 3.5% for maintenance) in oxygen (2 L/min for induction and 1 L/min for maintenance) (RWD Life Science Co., Ltd., Shenzhen, China, R540). The dorsal skin was shaved and disinfected using a betadine solution. A 2-cm longitudinal incision was created above the center of the T9–11 spinous processes. The muscles were carefully dissected, and the spinous processes were removed to expose the T10 lamina. The rats in the sham group were subjected to laminectomy only. A modified impactor was used to induce a contusive SCI model. The T9 and T11 vertebrae were stabilized using clamps, and the stereotaxic frame was adjusted to tightly center the impactor tip on the T10 spinal cord surface. The weight height was set at 50 mm. The pulling rod was removed, and the impactor tip was allowed to impact the spinal cord for 5 s. The rats’ tails and bilateral hind limbs twitched. Subdural congestion was observed in the impact region after removing the impactor tip. After the surgical incisions were sutured, the rats received postoperative care, including an intramuscular injection of penicillin (50,000 U/kg/day) for 3 days and manual emiction twice daily until their automatic micturition function was re-established.

### Hindlimb Locomotor Function Measurements

Hind limb locomotor function was evaluated weekly after surgery using the Basso, Beattie, and Bresnahan (BBB) open-field locomotor test [24]. The BBB locomotor rating scale, which consists of 21 points, was used to quantify voluntary movement and body weight support. An increase in the BBB score indicated an improvement in motor function.

### m6A Modification Microarray Assay

Total RNA from six spinal cord samples, including three SCI and three sham group samples, were extracted using TRIzol reagent (Invitrogen, Carlsbad, CA, USA) and quantified using a NanoDrop ND-1000 (Thermo Fisher Scientific, Waltham, MA, USA). Sample preparation and microarray hybridization were performed based on Arraystar’s standard protocols. Briefly, the six RNA samples were immunoprecipitated using an anti-m6A antibody (Synaptic System, Goettingen, Germany). The modified RNAs were eluted from immunoprecipitated magnetic beads and denoted as “IP,” and the RNA without m6A modification was recovered from the supernatant and denoted as “Sup.” The “IP” and “Sup” RNAs were labeled with Cy5 and Cy3, respectively, and amplified as cRNAs using the Arraystar Super RNA Labeling Kit. Then, the Arraystar rat mRNA and lncRNA epitranscriptomic arrays (8 × 60 K, Arraystar) were used to combine and hybridize the Cy3- and Cy5-labeled cRNAs at 65 °C for 17 h in an Agilent hybridization oven. After washing the slides, the arrays were scanned using an Agilent Scanner G2505C (Agilent, Santa Clara, CA, USA).

### Microarray Data Analysis

The acquired raw data were extracted and analyzed using Agilent Feature Extraction software (version 11.0.1.1). The raw intensities of IP (immunoprecipitated, Cy5-labeled) and Sup (supernatant, Cy3-labeled) samples were normalized using the average of the log2-scaled spike-in RNA intensities. m6A methylation and mRNA expression levels were calculated based on the percentage of modified and unmodified IP and Sup normalized intensities, respectively. The fold change and *P*-value between the SCI and sham groups were calculated. The RNAs with a cutoff of 1.5-fold change and *p* < 0.05 were defined as the differentially m6A-modified RNAs or expressed RNAs between the two groups.

### GO and KEGG Functional Enrichment Analyses

The differentially m6A-methylated and expressed (DME) mRNAs were selected for Gene Ontology (GO) and Kyoto Encyclopedia of Genes and Genomes (KEGG) pathway analyses using the clusterProfiler R package. The cis-regulated target genes predicted the function of lncRNA, which were derived from the mRNAs within 100 kb upstream and downstream of the lncRNAs. A *p*-value cutoff < 0.05 was used to determine the significantly enriched GO terms and pathways.

### Protein–Protein Interaction (PPI) Network Construction

The PPIs of DME mRNAs were identified using the STRING database (https://www.string-db.org/). DME mRNAs with interactions with combined scores greater than 0.4 were selected to construct a PPI network and visualized using Cytoscape software (version v3.7.1). Hub genes were identified based on the enrichment score of the MCODE plugin in Cytoscape.

### Methylated RNA Immunoprecipitation (MeRIP)

We performed a MeRIP assay using a Magna MeRIP™ m6A Kit (Millipore, Billerica, MA, USA) according to the manufacturer’s protocol. The total RNA samples were immunoprecipitated with an anti-m6A antibody (Synaptic Systems, Gottingen, Germany). The supernatant RNA was labeled “Sup,” and the IP RNA was labeled “MeRIP.” “IP” RNAs were used for first-strand cDNA synthesis using a SuperScript First-Strand Synthesis Kit (Invitrogen, Carlsbad, CA, USA). Immunoprecipitated RNA was analyzed using qRT-PCR and normalized with the input RNA. All primers used in this study are listed in Table S1. All experiments were performed in triplicate.

### RNA Extraction and RT-qPCR

TRIzol Reagent (Invitrogen, Carlsbad, CA, USA) was used to extract total RNA from all Sham and SCI group. A Hifair™ II 1st-Strand cDNA Synthesis SuperMix Kit (Yeasen Biotechnology, Wuhan, China) was used to synthesize the cDNA. A Hieff UNICON® Universal Blue qPCR SYBR Green Master Mix (Yeasen Biotechnology) was used for real-time quantitative polymerase chain reaction (RT-qPCR), and glyceraldehyde 3-phosphate dehydrogenase (*GAPDH*) was the endogenous control. The primer sequences used in this study are shown in Table S1.

### RNA Dot Blot

Thaw the RNA sample and measure its concentration on an ice-bound platform. Unify the sample concentration, dilute the sample continuously in a new enzyme-free tube, and mix well; the final concentrations of RNA were 400, 200, 100, and 50 ng/μL. Diluted samples are added to the membrane, which is then crosslinked by exposing it to a 254-nm UV lamp. The membrane is cleaned with TBST and incubated with blocking solution, primary antibody (m6A antibody 680551-lg, 1:1000), and secondary antibody (HRP-goat anti-mouse secondary antibody, 1:2000). After incubation, the membrane is exposed to ECL substrate and photographed. Finally, the membrane is placed in methylene blue staining buffer and washed with ddH_2_O to obtain a clean background. White light images are acquired as a load control after staining.

### Statistical Analysis of Data

Experiments were performed independently at least three times, and all data are expressed as mean ± standard deviation. The Student’s *t*-test was used to assess significant differences between sham and SCI group, and values of *p* < 0.05 were considered statistically significant.

## Results

### Establishment of a Rat Spinal Cord Injury (SCI) Model

**Fig. 1:**
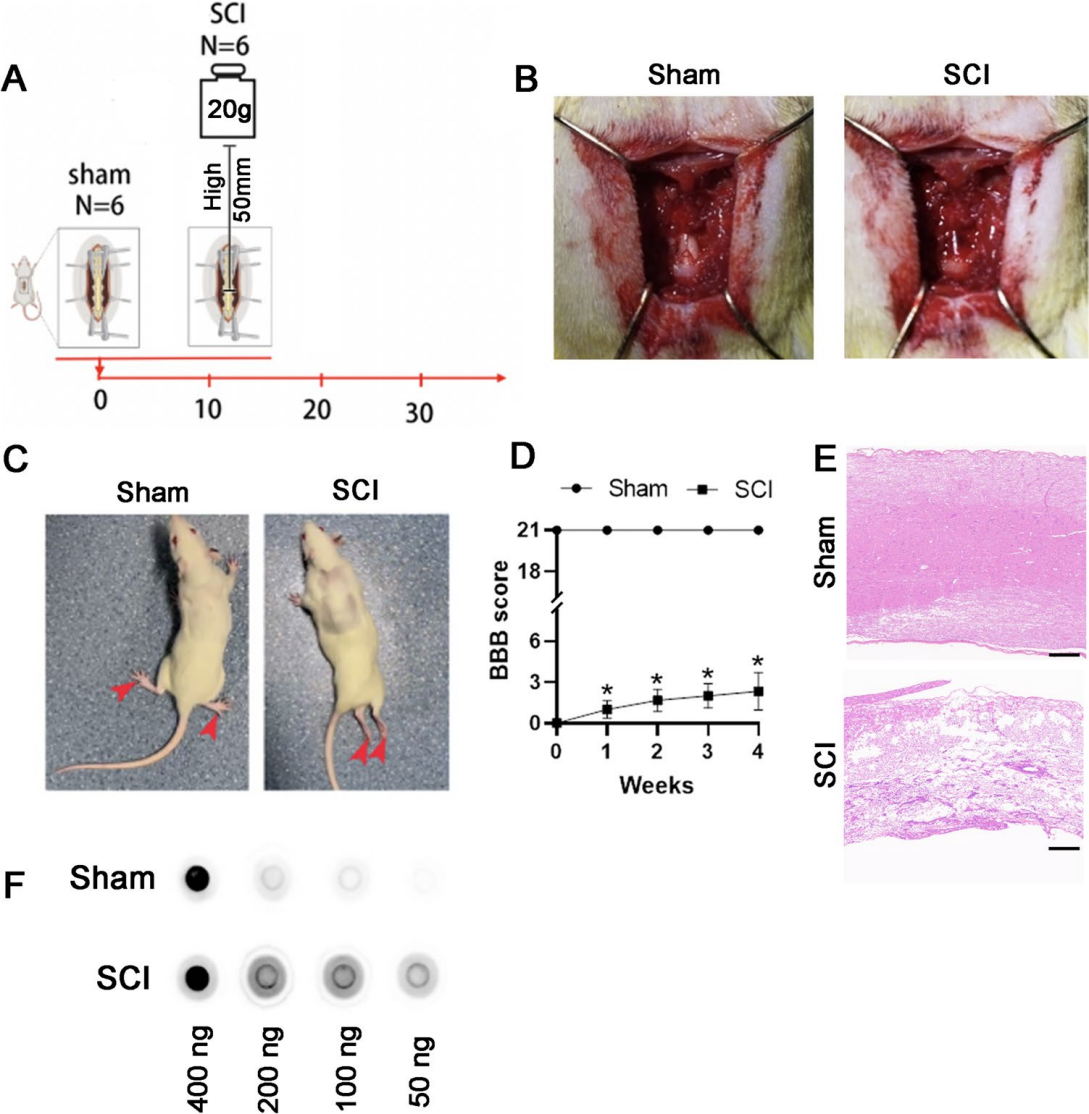
Construction of the rat spinal cord injury (SCI) model and the pathological changes after SCI. **A** Schematic diagram of SCI in the rat. Twelve rats were randomly divided into Sham and SCI groups. **B** Dorsal view of representative spinal cords from the Sham and SCI groups after surgery. Subdural congestion was observed in the SCI group. **C** These images depict the movement of rats’ feet (arrowheads) during an open field test performed 4 weeks after surgery in the Sham (left) and SCI (right) groups. **D** Graph of the BBB score of the hindlimb locomotor function in the two groups (*n* = 6/group; Student’s *t*-test, * p < 0.05). **E** Hematoxylin and eosin staining of spinal cord tissues of the Sham and SCI rats after 4 weeks of injury. Scale bars, 250 μm. **F** Total RNA was isolated from the spinal cords of SCI or sham surgery groups, and the changes in m6A modification levels before and after spinal cord injury were evaluated using  dot-blot. The 400 ng, 200 ng, 100 ng, and 50 ng refers to the amount of RNA loaded

The rats received a central thoracic spinal cord contusion at the T10 level to generate the SCI model, whereas the sham-operated rats only underwent laminectomy and were used as controls (Fig. 1A). SCI rats exhibited subdural congestion in the impact region after contusion compared with sham-operated rats (Fig. 1B). To further evaluate the success of the SCI model, we used the BBB to evaluate the locomotor function of the rats. We observed that the rats experienced hind limb paralysis immediately after SCI and showed little recovery of motor function for up to 4 weeks following injury (Fig. 1C, D). Hematoxylin and eosin staining was performed to further explore the effects of SCI. Four weeks after SCI, the morphology of the spinal cord was destroyed, motor neurons were significantly lost, and many glial cells were observed at the lesion site (Fig. 1E). These data indicate the successful construction of the SCI rat model. Dotblot showed that the level of m6A modification in spinal cord tissue significantly increased after chronic SCI (Fig. 1F), indicating that m6A modification is involved in the pathological process of SCI.

### Transcriptome Profile of m6A Methylation in SCI Rats

**Fig. 2:**
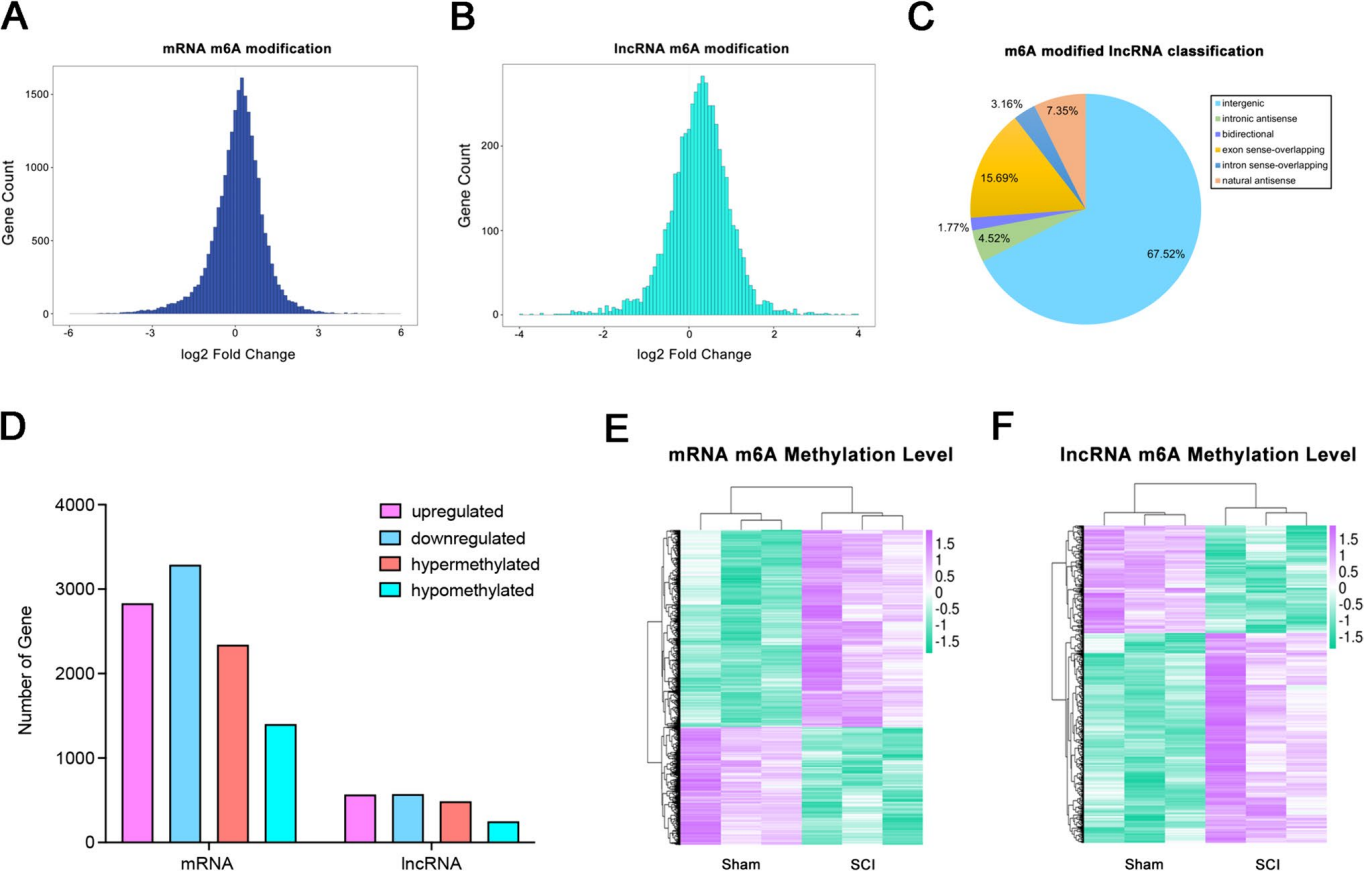
Alterations of the m6A modification profiles of mRNA and lncRNA in the spinal cord of the SCI rat model. Frequency histogram of the m6A-methylation modified mRNAs (**A**) or lncRNAs (**B**). **C** The classification of m6A-methylation modified lncRNAs. **D** The number of mRNAs and lncRNAs with differential m6A methylation or differential expressions. Hierarchical clustering analysis of the differentially m6A-methylated mRNAs (**E**) or lncRNAs in SCI group and Sham group (**F**). The m6A methylation levels are described in terms of a color scale, and the colors from green to purple indicated the intensity expression. The rows indicating differentially m6A-methylated transcripts and columns indicating samples

To investigate the role of m6A methylation in SCI, we performed mRNA and lncRNA m6A-methylation analyses by epitranscriptomic microarray in severely injured spinal cord tissues (SCI group) and sham-operated rat spinal cord tissues (sham group). In total, 22,284 m6A-modified mRNAs and 5132 m6A-modified lncRNAs were detected (Fig. 2A, B). Six types of lncRNAs were identified; the highest proportion of lncRNA types was originated from intergenic regions (67.52%) (Fig. 2C). Based on a screening threshold of fold-change ≥ 1.5 and *p* < 0.05, the differentially expressed genes and differentially m6A-methylated genes were identified. Compared to the sham group, 2833 mRNAs and 568 lncRNAs were significantly upregulated in the SCI group, and 3290 mRNAs and 574 lncRNAs were significantly downregulated (Fig. 2D, Table S2). Also, in the SCI group, 2343 mRNAs and 488 lncRNAs were hypermethylated, and 1402 mRNAs and 250 lncRNAs were hypomethylated compared to the sham group (Fig. 2D, E, F, Table S3).

### Functional Analyses of Differentially m6A-Modified mRNAs

**Fig. 3:**
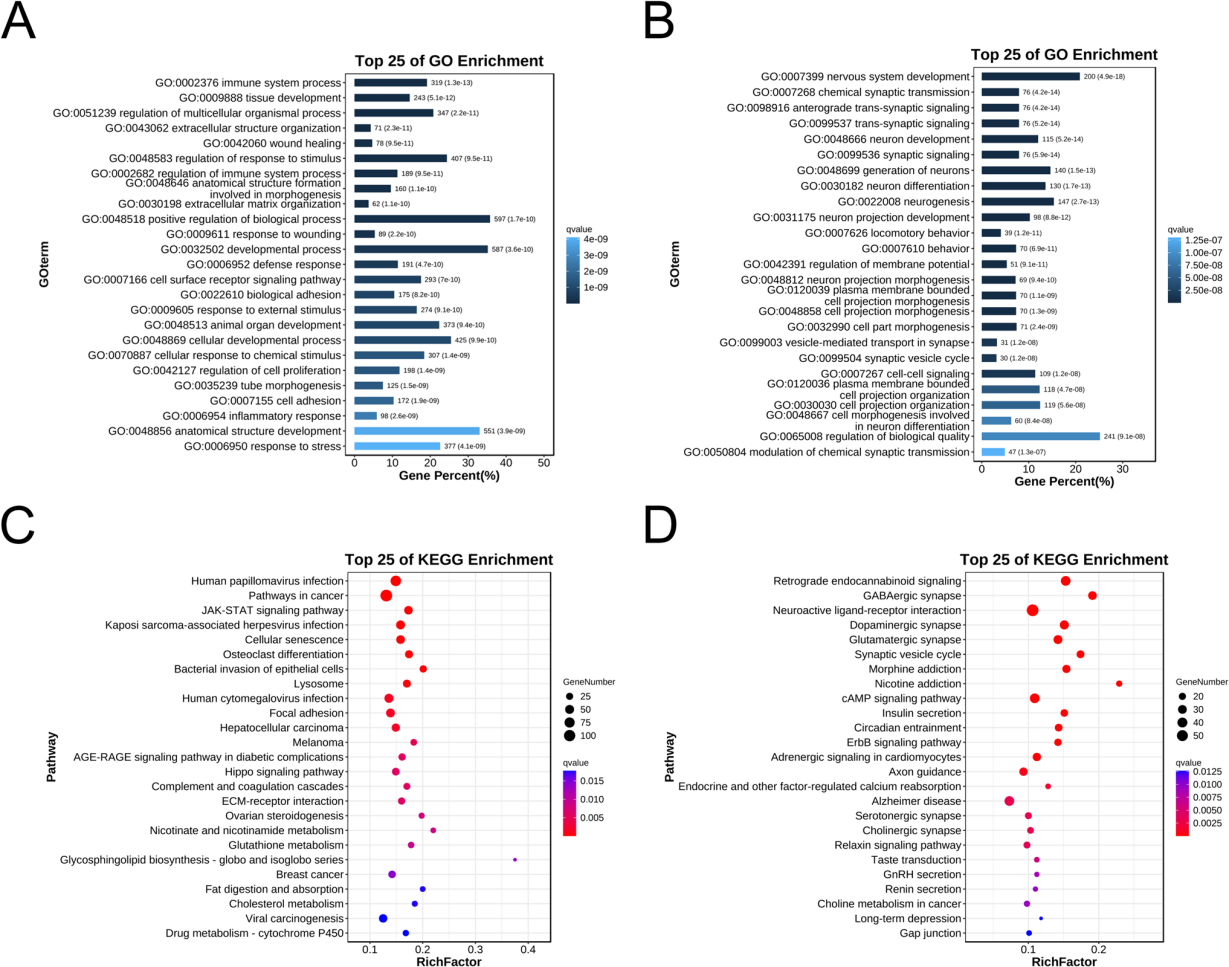
Biological function and pathway predictions of significantly differentially m6A-modified mRNAs in the spinal cord of SCI and Sham rats. **A** Top 25 Gene Ontology-Biological Process (GO-BP) terms enriched with m6A-hypermethylated mRNAs. **B** Top 25 GO-BP terms enriched with m6A-hypomethylated mRNAs. **C** Top 25 Kyoto Encyclopedia of Genes and Genomes (KEGG) pathways enriched with m6A-hypermethylated mRNAs. **D** Top 25 KEGG pathways enriched with m6A-hypomethylated mRNAs

GO and KEGG pathway enrichment analyses were performed to clarify the potential functions of the differentially m6A-modified transcripts after SCI. GO biological analysis showed that the hypermethylated mRNAs were mainly enriched in the immune system process, extracellular matrix organization, and inflammatory response (Fig. 3A, Table S4). In contrast, the hypomethylated mRNAs were associated with nervous system development, trans-synaptic signaling, and neuronal development (Fig. 3B, Table S4). KEGG pathway analysis indicated that the hypermethylated mRNAs participated in cellular senescence, focal adhesion, and complement and coagulation cascades (Fig. 3C, Table S4). Conversely, the hypomethylated mRNAs were involved in GABAergic synapse, neuroactive ligand-receptor interaction, and cAMP signaling pathway (Fig. 3D, Table S4).

### Functional Analyses of Differentially m6A-Modified lncRNAs

**Fig. 4:**
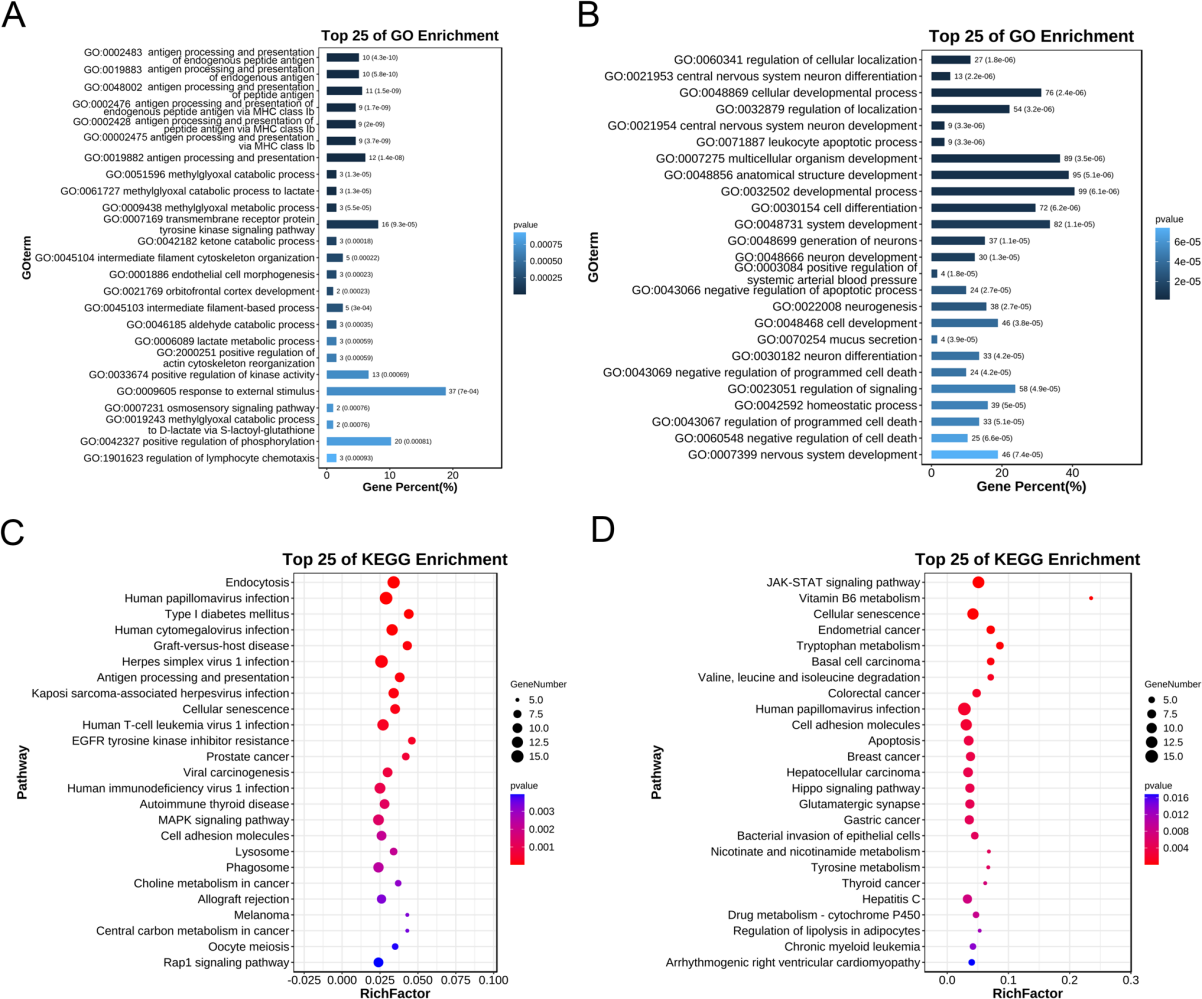
Biological function and pathway predictions of significantly differentially m6A-modified lncRNAs in the spinal cords of SCI and Sham rats. **A** Top 25 GO-BP terms enriched with m6A-hypermethylated lncRNAs. **B** Top 25 GO-BP terms enriched with m6A-hypomethylated lncRNAs. **C** Top 25 KEGG pathways enriched with m6A-hypermethylated lncRNAs. **D** Top 25 KEGG pathways enriched with m6A-hypomethylated lncRNAs

Based on the cis-target differentially m6A-modified mRNAs of the lncRNAs, we predicted the functions of the differentially m6A-modified lncRNAs. We observed that the hypermethylated lncRNAs were mainly enriched in the GO terms, including antigen processing and presentation, intermediate filament cytoskeleton organization, and positive regulation of kinase activity (Fig. 4A, Table S5). Conversely, the hypomethylated lncRNAs were associated with central nervous system neuron differentiation, negative regulation of apoptotic processes, and homeostatic processes (Fig. 4B, Table S5). Moreover, KEGG pathway analysis indicated that hypermethylated lncRNAs participated in antigen processing and presentation, the MAPK signaling pathway, and cell adhesion molecules (Fig. 4C, Table S5), whereas hypomethylated lncRNAs were involved in the JAK-STAT signaling pathway, cellular senescence, and apoptosis (Fig. 4D, Table S5).

### Integration Analysis of mRNA m6A-Methylation and Gene Expression

**Fig. 5:**
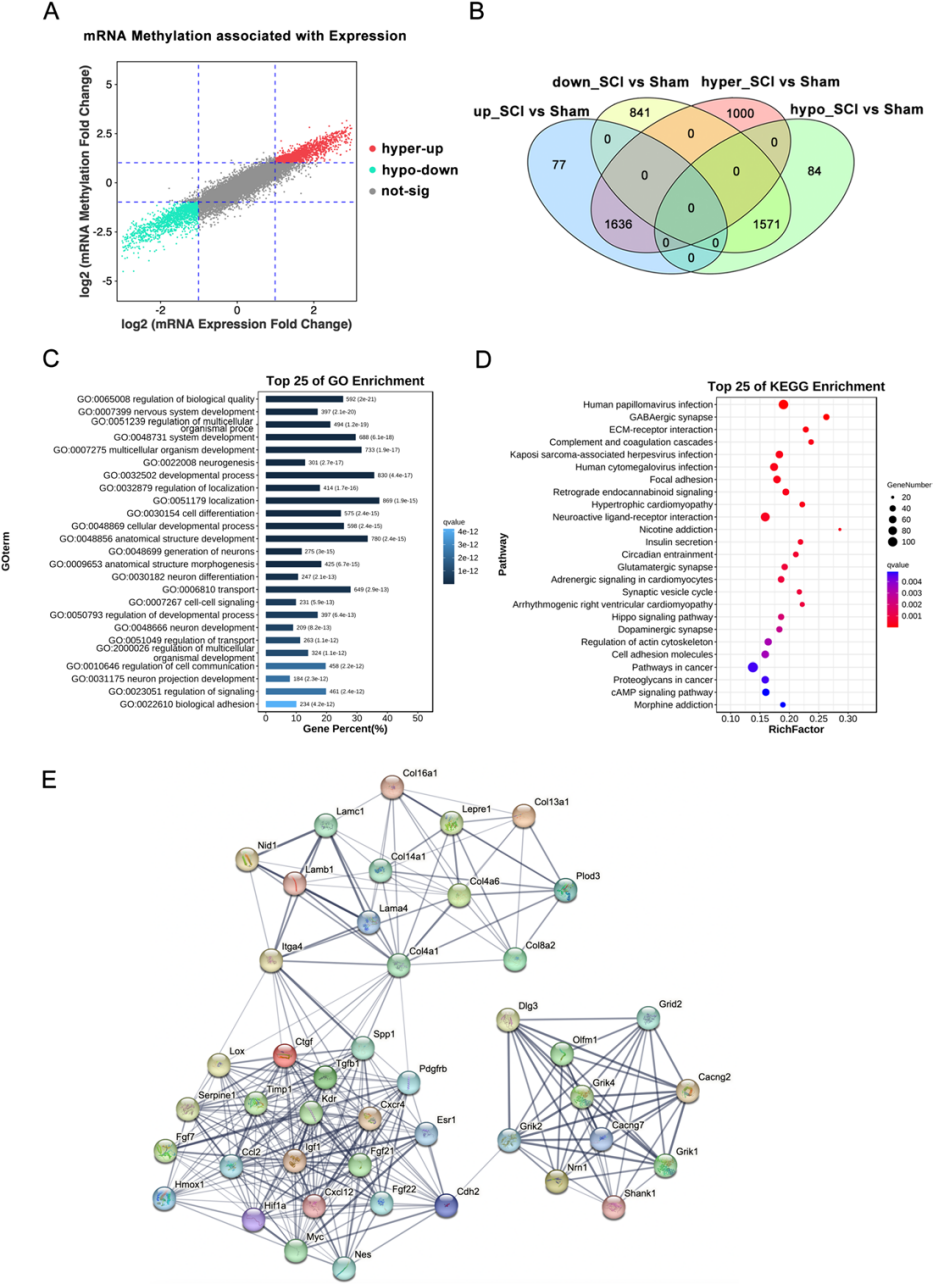
Conjoint analysis of mRNA m6A modification and mRNA expression. **A** Nine-quadrant graph showing the correlation between the m6A modification level and transcriptome expression level of differentially m6A-methylated and expressed (DME) mRNAs. Each dot represents a gene; the red dots indicate hyper m6A methylation and high transcriptome expression, the green dots indicate hypo m6A methylation and low transcriptome expression, and the gray dots indicate non-differential genes. The threshold range we set was |log2Fd|≥ 1 (|fold change|≥ 2). **B** Venn diagram of differentially m6A-methylated and differentially expressed mRNAs. **C** Top 25 GO-BP terms enriched with DME mRNAs. **D** Top 25 KEGG pathways enriched with DME mRNAs. **E** Protein–protein interaction network and hub gene identification of DME mRNAs based on the MCODE plugin in Cytoscape

To characterize the correlation between m6A modification and expression, differentially expressed and m6A-methylated mRNAs and lncRNAs (fold change ≥ 2) were intersected. Two modes of interaction were identified for the mRNAs (Fig. 5A, B, Table S6): m6A hypermethylation with upregulated transcription levels (hyper-up, 1636 mRNAs) and m6A hypomethylation with downregulated transcription levels (hypo-down, 1571 mRNAs). Functional analysis demonstrated that the DME mRNAs were mainly associated with the GO terms, including nervous system development, cell differentiation, and biological adhesion, and were mainly involved in GABAergic synapses, ECM-receptor interactions, and focal adhesion pathways (Fig. 5C, D, Table S7). To identify the hub genes that may play critical roles mediated by m6A methylation, we analyzed all DME mRNAs using PPI data from the STRING database and the MCODE plugin in Cytoscape. The most enriched MCODE module had a score of 13.442, consisting of 44 nodes and 289 edges (Fig. 5E, Table S8).

### Integration Analysis of lncRNA m6A-Methylation and Gene Expression

**Fig. 6:**
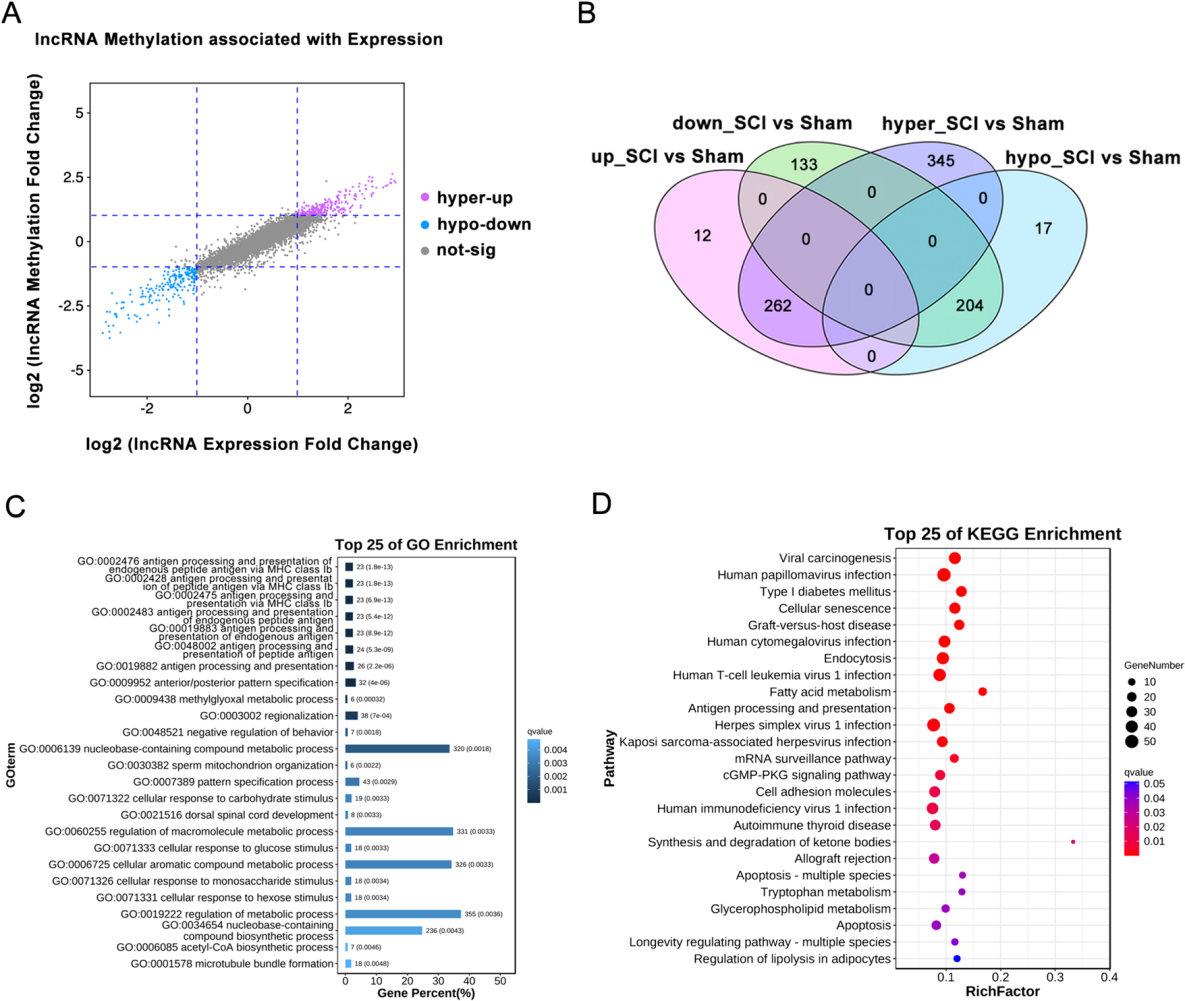
Conjoint analysis of lncRNA m6A modification and lncRNA expression. **A** Nine-quadrant graph showing the correlation between the m6A modification level and transcriptome expression level of the differentially m6A-methylated and expressed (DME) lncRNAs. Each dot represents a gene; the purple dots indicate high m6A methylation and high transcriptome expression, the blue dots indicate low m6A methylation and low transcriptome expression, and the gray dots indicate non-differential genes. The threshold range we set was |log2Fd|≥ 1 (|fold change|≥ 2). **B** Venn diagram of differentially m6A-methylated and differentially expressed lncRNAs. **C** Top 25 GO-BP terms enriched with DME lncRNAs. **D** Top 25 KEGG pathways enriched with DME lncRNAs

Two modes of interaction were identified for lncRNAs (Fig. 6A, Table S6): m6A hypermethylation with upregulated transcription levels (hyper-up, 262 lncRNAs) and m6A hypomethylation with downregulated transcription levels (hypo-down, 204 lncRNAs; Fig. 6B, Table S6). Functional analysis revealed that the DME lncRNAs were mainly associated with the GO terms, including antigen processing and presentation, dorsal spinal cord development, and regulation of metabolic processes, and were mainly involved in cellular senescence, the cGMP-PKG signaling pathway, and apoptotic pathway (Fig. 6C, D, Table S7). These results suggest a close relationship between *m6A* methylation and gene expression levels in the spinal cord tissues after SCI.

### Validation by MeRIP-qPCR and RT-qPCR

**Fig. 7:**
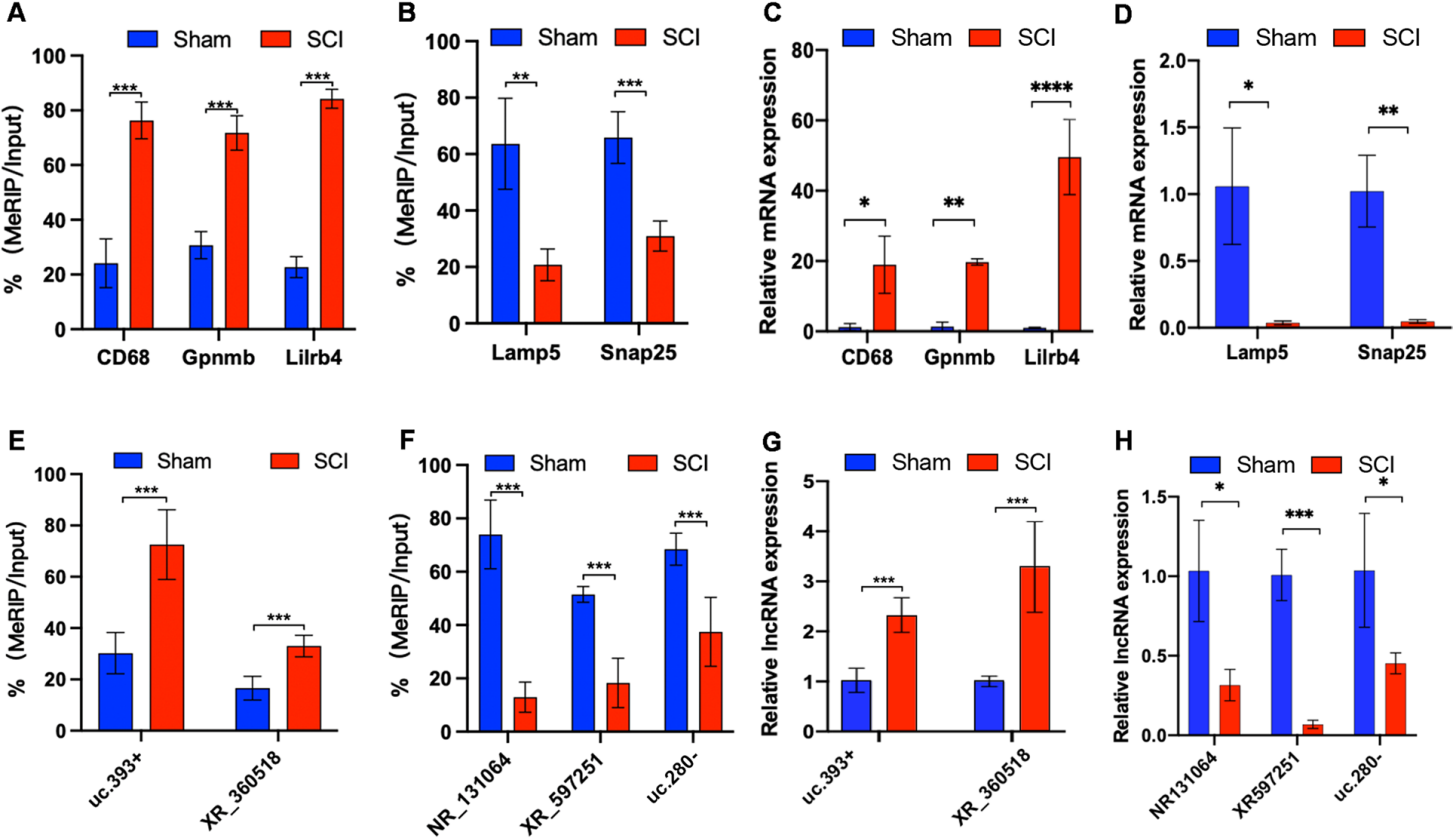
Validations of the m6A methylation level and expression level of the mRNAs and lncRNAs by MeRIP-qPCR and RT-qPCR. The m6A levels of hyper-up mRNAs (**A**) and lncRNAs (**E**), and the m6A levels of hypo-down mRNAs (**B**) and lncRNAs (**F**) were analyzed by MeRIP-qPCR; the expression level of hyper-up mRNAs (**C**) and lncRNAs (**G**), and the expression level of hypo-down mRNAs (**D**) and lncRNAs (**H**) were analyzed using qRT-PCR

In order to verify the accuracy of the sequencing results, the hyper-methylated with up-regulated (CD68, Gpnmb, Lilrb4) and hypo-methylated with down-regulated (Lamp5, Snap25) mRNAs and hyper-methylated with up-regulated (XR_360518, uc.393 +) and hypo-methylated with down-regulated (NR_131064, uc.280-, XR_597251) lncRNA were chosen for MeRIP-qPCR and qRT-PCR (Fig. 7). The results of them were consistent with the microarray results, indicating the reliability of the microarray data.

## Discussion

SCI is one of the most common and debilitating neurological injuries worldwide, resulting in severe disability. However, despite advances in surgical techniques, no effective treatment for this severely disabling disease is available [25]. After SCI, individuals experience a transition from the acute phase to the chronic phase, during which gene expression changes are also time dependent [26]. Therefore, further exploring the molecular mechanism and changes in the microenvironment after SCI are crucial for developing better treatment plans.

As the most prevalent RNA modification, m6A plays a critical role in SCI pathology [18, 19]. The global RNA m6A levels were decreased in mice with subacute SCI (3 days after SCI) in mice, and the low methylation level and high transcript level of genes related to neural regeneration are confirmed in zebrafish with acute SCI (24 h after SCI) [18, 19]. The study of the alterations in the m6A levels of lncRNA in SCI models is still very limited. Based on the microarray data of the spinal cord tissues from chronic SCI (30 days after SCI) and sham-operated rats, we analyzed the changes in mRNA and lncRNA m6A-methylated modification and expression levels in the SCI and sham groups and identified key pathways related to SCI. To verify the accuracy of the microarray results, we randomly selected five DME mRNAs and lncRNAs, respectively, for RT-qPCR and MeRIP-qPCR. Abundant differentially methylated and differentially expressed mRNAs and lncRNAs were identified in the SCI group compared with the sham group. A conjoint analysis of m6A methylation and expression of mRNAs and lncRNAs revealed a strong positive correlation. However, two reports indicated a negative correlation between m6A modification and expression levels in mice and zebrafish SCI models [18, 19]. We speculate that this may be related to the acute phase of SCI and the different species studied in these reports. These results provide new insights into the pathogenesis of SCI and new directions and molecular basis for the precise diagnosis and treatment of SCI in the future.

GO biological analysis showed that the hypermethylated mRNAs were mainly enriched in immune and inflammatory response processes and extracellular matrix organization, whereas the hypomethylated mRNAs were mainly involved in neuronal development, consistent with previous reports [27, 28]. After SCI, the interaction between the central nervous and immune systems is disrupted, leading to a significant systemic decline in immune function [29]. A series of complex immune cascade reactions occur after SCI, with neutrophils first arriving at the injury site and reaching their peak infiltration 24 h after the injury. Members of the matrix metalloproteinase family secreted by neutrophils in response to SCI promote the entry of cells (such as inflammatory cells) into the central nervous system by regulating the blood–brain barrier, thereby affecting the recovery of neurological functions after SCI [30–33]. This is consistent with the hypomethylated and upregulated gene expression levels of MMP9, MMP12, and MMP14 observed in this study. Subsequently, macrophages are recruited from the circulatory system and peak 7 days after injury, releasing inflammatory components, such as tumor necrosis factor (TNF) and interleukins (ILs), aggravating the secondary injury. In this study, Tnfsf9, Tnfsf13, and IL-18 bp were upregulated and hypermethylated, leading to TNF expression produced by macrophages in the injured spinal cord, polarizing M/Ms toward the M1 state, forming fibrous scars, and inhibiting axonal growth, which is not conducive to SCI repair [34–37]. Finally, lymphocytes invade the injured site via cytokines secreted by macrophages, participate in immune inflammation, and lead to immune microenvironmental disorders [38, 39].

T-cell infiltration has been observed in injured spinal cord tissues in various SCI animal models [40]. γδ T cells are the early sources of interferon (IFN)-γ, which are recruited to the SCI site through CCL2/CCR2 signaling, promoting inflammatory response and exacerbating nerve damage [41, 42]. In this study, the expression and m6A methylation levels of IFNgR1 and CCL2 increased after SCI. Although the immune response after SCI has many beneficial effects, the large-scale inflammatory response is a key factor hindering nerve regeneration and repair [43, 44].

SCI disrupts neuronal connections and causes trauma to various cells within the tissue. Studies have shown that transcriptions related to nervous system development after SCI are mainly enriched in cytoplasmic polyadenylation elements (CPE) [45]. The m6A methylation and expression levels of CPE-binding protein 1 (Cpeb1) decreased after SCI in our study, and its overexpression promoted axonal regeneration following injury [45].

In this study, microtubule-associated protein 2 (MAP2), an important gene involved in microtubule assembly in neurogenesis, demonstrated a significantly decreased expression and hypomethylated expression in the spinal cords of SCI rats [46]. One study has provided evidence that specific gait training can significantly improve the motor function of rats with incomplete SCI and increase the expression level of MAP2 in the motor cortex [46]. Moreover, the fibroblast growth factor (FGF) is a crucial regulator of spinal cord development; it also has a promising role in nerve injury therapy after SCI and is co-localized with MAP2 in neurons [47, 48]. FGF13 is enriched in axonal growth cones, highly expressed at an early stage after SCI, and gradually decreases at later stages after SCI [48]. Loss of FGF13 increases the branching and leading processes of axons and impairs neuronal polarization, whereas FGF13 upregulation facilitates functional recovery following SCI by promoting neuronal polarization, axon formation, and growth cone initiation [48, 49]. In addition, FGF13 mutant mice exhibit an imbalance in excitatory and inhibitory synaptic input activity within the local hippocampal circuit, leading to a clinical phenotype of epilepsy [50]. Decreased expression and hypomethylation of FGFR2 and FGF13 were observed in the SCI rats in our study. Contrary to our results, Chen et al. discovered increased FGFR2 expression after acute SCI, which may have been caused by different detection stages [51].

Functional analysis revealed that hypomethylated lncRNAs were mainly enriched in the apoptotic process, consistent with a previous report [52]. After SCI, apoptotic cells are observed in neurons, astrocytes, oligodendrocytes, and microglia, which may be the main cause of long-term neurological deficits [53, 54]. Under hypoxia, the expression level of the lncRNA TMEM235 in rat bone marrow mesenchymal stem cells (BMSCs) was significantly downregulated and was positively correlated with the degree of hypoxia and the apoptosis rate [55]. lncRNA TMEM235 was co-localized with the important anti-apoptotic protein BIRC5; the overexpression of lncRNA TMEM235 can inhibit the apoptosis of hypoxia-induced BMSCs and promote their survival by promoting BIRC5 expression [55]. In our study, lncRNA TMEM235 underwent m6A demethylation after SCI, and its expression was significantly reduced. Moreover, BIRC5 showed significantly hyper m6A-methylated and increased expression levels, but not after SCI. Furthermore, lncRNA PPP2R2B was co-located with the PPP2R2B gene, the hypermethylation of which can cause acquired apoptosis deficiency in systemic autoimmune diseases [56]. The m6A methylation and expression levels of lncRNA PPP2R2B were significantly downregulated in the SCI group, consistent with results obtained from completely transected SCI [57]. However, the molecular mechanism of action of lncRNA TMEM235 and PPP2R2B in SCI requires further research.

However, this study had some limitations. We mainly focused on m6A modification profiles and did not conduct functional experiments on the molecular mechanisms underlying m6A methylation. This should be clarified in future studies. In conclusion, we identified differential m6A modifications and expression profiles of mRNAs and lncRNAs after SCI in rats. The possible roles of m6A modification in SCI were predicted by bioinformatics analysis. Our results suggest that m6A modification may be involved in the pathogenesis of SCI, and the identification of key pathways may provide new clues and evidence for developing more effective therapeutic targets for SCI.

## Supplementary Information

Below is the link to the electronic supplementary material.

Supplementary file1 (XLSX 10 KB)

Supplementary file2 (XLSX 1376 KB)

Supplementary file3 (XLSX 940 KB)

Supplementary file4 (XLSX 720 KB)

Supplementary file5 (XLSX 411 KB)

Supplementary file6 (XLSX 695 KB)

Supplementary file7 (XLSX 775 KB)

Supplementary file8 (XLSX 626 KB)

## Data Availability

The data and materials used in this study will be made available to the research community upon reasonable request. The relevant datasets are stored in publicly accessible databases. Additionally, all experimental materials and detailed descriptions of the experimental methods used in this study can be found in the Supplementary Materials. If you require further information or have any questions, please contact me at liukai_qing@163.com.
